# Extrahepatic Biliary Papillomatosis in a Child

**Published:** 2013-09-21

**Authors:** A Singh, M Bajpai, N Sharma, M Jana

**Affiliations:** Department of Paediatric Surgery,All India Institute of Medical Sciences (AIIMS), New Delhi-110029, India.; Department of Paediatric Surgery,All India Institute of Medical Sciences (AIIMS), New Delhi-110029, India.; Department of Paediatric Surgery,All India Institute of Medical Sciences (AIIMS), New Delhi-110029, India.; Department of Radiology,All India Institute of Medical Sciences (AIIMS), New Delhi-110029, India.

**Keywords:** Extrahepatic biliary papillomatosis, Common bile duct, Polyp

## Abstract

In children, benign neoplasms of extrahepatic biliary ducts are extremely rare. We report a case of 3 year old girl who presented with abdominal pain and jaundice for 6 months. The final diagnosis on histopathology was papillomatosis in lower common bile duct.

## INTRODUCTION

Benign neoplasms of extrahepatic biliary ducts are infrequent in children. Their importance lies in their ability to mimic malignant lesions in such locations. Commonest presenting symptoms are pain, jaundice and acute cholangitis. Diagnosis of these cases predominantly is intraoperative.[1-3] This case highlights the above observations during management of the index patient.

## CASE REPORT

A 3-year-old female child presented with the complaints of pain in upper abdomen along with jaundice and fever for last six months. Abdominal examination was unremarkable. With the clinical possibility of a choledochal cyst, ultrasonography (USG) along with liver functions and routine haematological investigations were advised. USG showed impacted worm like appearance in the lower common bile duct with proximal dilatation (Fig. 1). In view of deepening jaundice child was advised endoscopic retrograde cholangiopancreatography (ERCP) to clear the worm load in lower end of common bile duct (CBD) and to place the stent for establishing the bile flow. Endoscopic sphincterotomy with placement of 10 Fr stent was done but worm could not be cleared. Child also received antihelminthic drugs. Following stent placement child recovered from jaundice within two weeks. As there was a doubt of persistent worm in the distal CBD, exploration was planned and after obtaining informed written consent, child was explored under general anaesthesia. Through right upper transverse incision abdomen was explored. CBD was traced using stent as a guide. On opening the CBD, multiple polyps were found with stent in situ (Fig. 2). No stone or worm was found. In view of possible malignancy a frozen section was sent and when confirmed to be benign, polyps were removed completely and CBD repaired over a T-tube. Stent was also removed. Postoperatively T-tube was removed after 3 weeks. Postoperatively child recovered uneventfully and presently she is in follow up and doing well. Histopathology of the resected specimen was reported as papillomatosis of the CBD.

**Figure:**
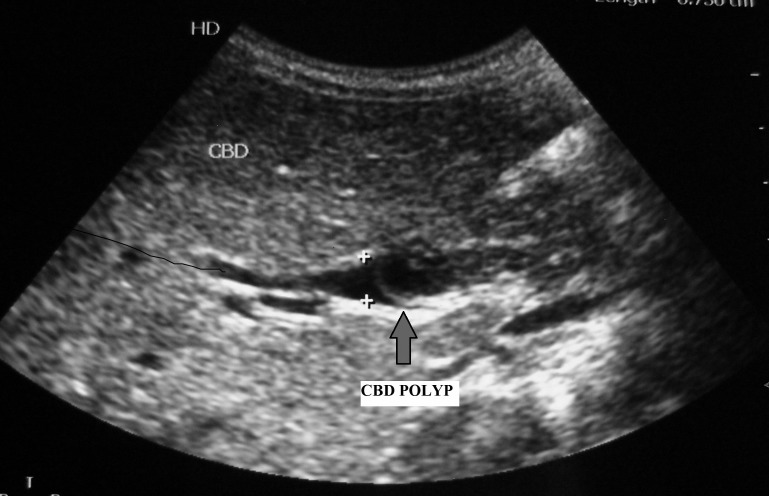
Figure 1:Ultrasound showing filling defect in the lower CBD (polyp).

**Figure:**
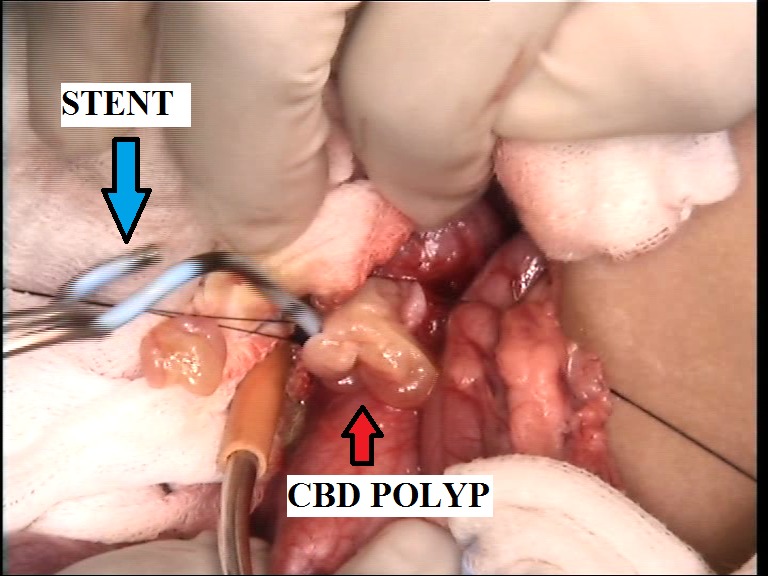
Figure 2: Intraoperative photograph showing CBD polyp and stent.

## DISCUSSION

Benign tumors and tumor like lesions from the gallbladder and bile duct have a wide spectrum and despite this diversity these lesions share common embryologic origins and histologic characteristics. Benign neoplasms of epithelial origin are adenomas, cystadenomas and the rarely multiple polyps may be found as in present case of biliary papillomatosis. Granular cell tumors, neurofibromas, ganglioneuromas, paragangliomas and leiomyomas are examples of benign tumors that may originate from nonepithelial structures.

Biliary papillomatosis is rare with only 140 cases reported in literature and that too in adults.[1] Chappet in 1894 reported the first case of biliary papillomatosis.[2] The malignant potential of benign polypoid lesions of the extrahepatic biliary system is controversial and not fully understood. The current literature supports the notion of malignant transformation of these benign epithelial lesions.[3-5] Reported male: female ratio is 2:1 with peak incidence in 4th decade of life.[3,4] Commonest presenting symptoms are pain, jaundice and acute cholangitis as in our case.[3,4,6] Diagnosis of these cases predominantly is intraoperative with few cases being suspected on ultrasound.[7] In present case it was discovered after CBD exploration. To conclude, although rare, biliary papillomatosis should be kept in mind when examining a patient with obstructive jaundice, acute cholangitis and abdominal pain in children.

## Footnotes

**Source of Support:** Nil

**Conflict of Interest:** None declared

